# Application of Steel Slag as an Aggregate in Concrete Production: A Review

**DOI:** 10.3390/ma16175841

**Published:** 2023-08-25

**Authors:** Zhengyi Ren, Dongsheng Li

**Affiliations:** 1 School of Civil Engineering, Dalian University of Technology, Dalian 116024, China; 12006035@mail.dlut.edu.cn; 2 State Key Laboratory of Coastal and Offshore Engineering, Dalian University of Technology, Dalian 116024, China

**Keywords:** steel slag aggregate, expansion mechanism, treatment method, concrete, mechanical properties, durability

## Abstract

Steel slag is a solid waste produced in crude steel smelting, and a typical management option is stockpiling in slag disposal yards. Over the years, the massive production of steel slags and the continuous use of residue yards have led to vast occupation of land resources and caused severe environmental concerns. Steel slag particles can potentially be used as aggregates in concrete production. However, the volume stability of steel slag is poor, and the direct use of untreated steel slag aggregate (SSA) may cause cracking and spalling of concrete. The present research summarizes, analyzes, and compares the chemical, physical, and mechanical properties of steel slags. The mechanism and treatment methods of volume expansion are introduced, and the advantages, disadvantages, and applicable targets of these methods are discussed. Then, the latest research progress of steel slag aggregate concrete (SSAC) is reviewed. Using SSA leads to an increase in the density of concrete and a decrease in workability, but the mechanical properties and durability of SSAC are superior to natural aggregate concrete (NAC). Finally, future research in this field is proposed to motivate further studies and guide decision-making.

## 1. Background

Concrete is the most-used building material globally, with global consumption of about 12 billion tons annually [1]. Concrete generally comprises 10–20% cement, 70–80% natural aggregate (NA), and 5–10% water by mass [2]. The massive demand for concrete has led to the overexploitation of high-quality river sand and gravel. Resource depletion has become an increasing concern. Besides, high-quality river sand and gravel are local resources commonly distributed unevenly. The contradictions between the supply and demand of river sand and gravel are becoming increasingly severe, resulting in the concrete industry facing widespread resource constraints. As it is impossible to eliminate the use of concrete, the sustainable development of the concrete industry must focus on finding alternatives to NAs as a solution to the current problems, while taking into account the environment, resources, and economic factors in the decision-making stage.

**Figure 1:**
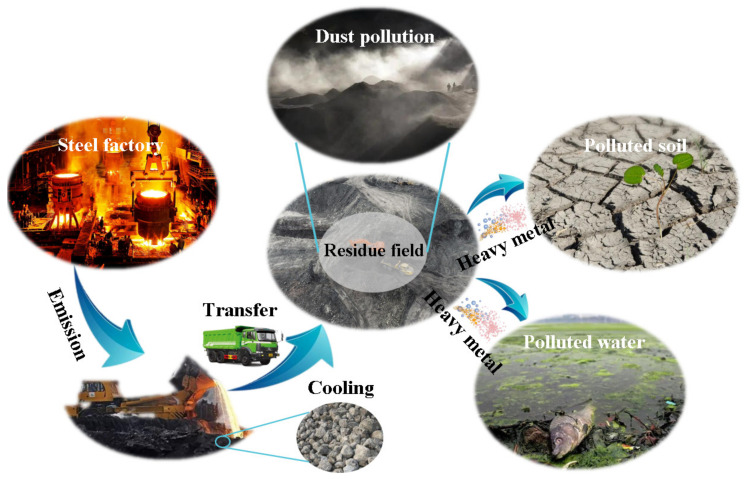
Environmental threat of unutilized steel slag.

Steel slag is the byproduct of the crude steel smelting process, and the discharge is approximately 12–20% of crude steel production [3]. The annual data released by the World Steel Association shows that the global crude steel production in 2022 is 1878.5 million tons [4]. Conservative estimates of steel slag emissions exceed 225 million tons. Take China as an example; as the world’s largest producer of crude steel, China’s steel slag inventory in 2012 exceeded 400 million tons [5], reached 1468 million tons in 2020 [6], and is still increasing, at a rate of more than 100 million tons per year [7]. Unutilized steel slags are commonly landfilled or stockpiled in slag disposal yards, which occupy precious land resources and lead to severe environmental threats [8], as shown in Figure 1. Tightening legislation and increasingly strict environmental criteria have incentivized attempts to recycle and reuse steel slag. Among them, the research in concrete production has made satisfactory progress.

Steel slag could be valuable for promoting a sustainable relationship between the concrete and steel industries. C_2_S, C_3_S, C_4_AF, and C_3_A in steel slags are typical mineral phases of Portland cement, indicating the potential of steel slags to function as hydraulic materials. Therefore, one recycling option is to grind steel slags into a fine powder and use it as a supplementary cementitious material. It has been proved that replacing cement with 10–20% steel slag powder does not weaken the mechanical strength of the system [9,10,11], but extends the setting time of the mixed slurry [12]. Steel slag can also act as a corrective raw material for iron in the cement production process due to the high content of iron oxides. However, steel slags have the disadvantage of poor grinding performance [13]. Grinding steel slag particles into fine powder will cause additional grinding energy consumption and increase the mill fault ratio. Higher equipment maintenance and replacement cost significantly limit the use of steel slag as supplementary cementitious material and cement raw material.

Another recycling option is to sieve steel slag into a good grading range, then use it as a coarse/fine aggregate to produce concrete. This option is the most likely option to realize the bulk utilization of steel slag, which is significant in alleviating the resource and environmental dilemma of the concrete and steel industries. However, the volume stability of steel slag aggregate (SSA) is poor, and the prepared concrete has the risk of cracking [14]. To date, commonly used methods for modifying the volume stability of SSA include natural aging [15], accelerated carbonation [16], melting slag converting [17], and external constraint [18]. These methods have made significant progress in recent years. The present paper reviews the production, properties, and treatment method of SSA. Then, the properties of SSAC, including density, workability, mechanical properties, and durability, are collected, analyzed, and summarized. Finally, the current research gaps are outlined, and future research needs are identified.

## 2. Characteristics of Steel Slag

### 2.1. Production of Steel Slag

**Figure 2:**
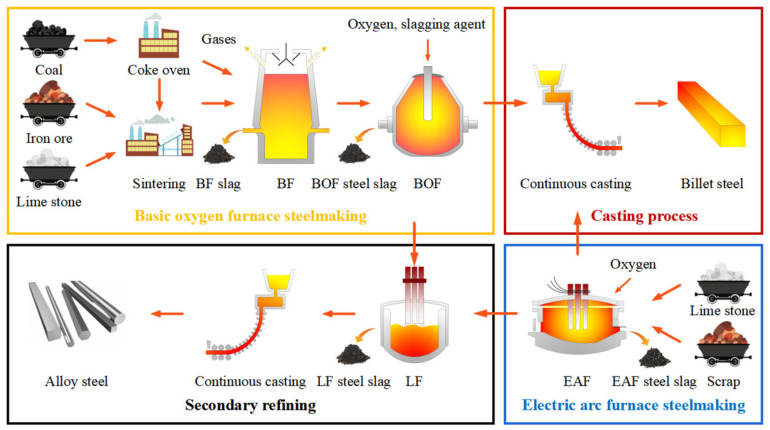
Flowchart of the smelting process.

**Figure 3:**
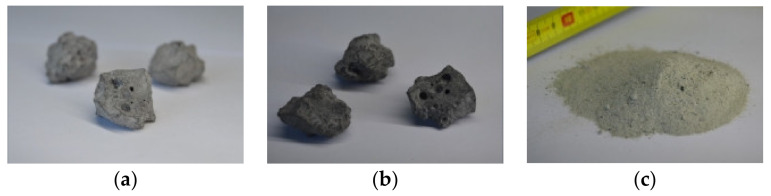
Typical morphology of steel slag [19]. (**a**) BOF steel slag. (**b**) EAF steel slag. (**c**) LF steel slag.

Slags of various types account for approximately 90% (by mass) of the byproducts discharged from the crude steel smelting process; the others are gas, dust, and sludge [19]. Figure 2 presents the crude steel smelting process. According to the difference in crude steel smelting processes, steel slag can be divided into basic oxygen furnace (BOF) steel slag, electric arc furnace (EAF) steel slag, and ladle furnace (LF) steel slag. The typical morphologies of different types of steel slag are shown in Figure 3.

BOF steel slag is the solid waste produced in the converter steelmaking process, and its discharge is approximately 100–150 kg per ton of BOF steel [20,21,22,23]. BOF steel slag commonly has a grayish-white appearance and a vesicular nature, with many non-interconnected pores [24]. Converter steelmaking, which typically uses iron ore as a raw material, accounts for over two-thirds of the world’s crude steel production. Therefore, the output of BOF steel slag accounts for about 70% of the total steel slag production [25].

EAF steel slag is the byproduct of electric furnace steelmaking, the output of which is approximately 110–200 kg per ton of EAF steel [15,19,23,26,27]. Electric furnace steelmaking is suitable for making high-quality steel, and the primary raw material is recycled scrap steel. EAF steel slag is slightly darker and appears grayish or grayish-black. The raw materials and principles of EAF steel slag and BOF steel slag are similar, and the differences in morphology and structure of the two kinds of slag are limited.

LF steel slag is the solid waste produced by the secondary refining of BOF and EAF steel. About 10–50 kg of LF steel slag is discharged for each ton of crude steel refined [11,27,28,29]. Simultaneously, LF steel slag appears as a loose powder, which is more suitable for utilization as a supplementary cementitious material than the aggregate. Therefore, this study has not focused on its evaluation. In the following discussions, steel slag only represents BOF and EAF steel slags, unless otherwise specified.

### 2.2. Chemical Composition of Steel Slag

Table 1 shows the extensively collected data from the literature covering five years. In general, steel slag is mainly composed of different oxides, and the concentration of CaO, SiO_2_, Fe_x_O_y_ (representing Fe, FeO, Fe_2_O_3_, and Fe_3_O_4_), Al_2_O_3_, and MgO is nearly 90%, while the rest covers the oxides of P, Mn, S, Ti, Na, K, Sr, and others. The chemical compositions of BOF and EAF steel slags are somewhat similar; despite this, the predominance of higher contents of specific components can be analyzed, due to their different steelmaking processes. BOF steel slag has a higher CaO content, while EAF steel slag has higher SiO_2_, Fe_x_O_y_, and Al_2_O_3_ concentrations. Furthermore, oxides of Cr, Pb, V, Mn, Zn, and other heavy metals can be observed in steel slag [30]. In the smelting process of EAF steel, alloying elements must be added, resulting in a higher content of heavy metal oxides [31,32].

There are more than ten kinds of mineral phases in steel slag that can be observed. The most common mineral phases in BOF steel slag are larnite (C_2_S), alite (C_3_S), brownmillerite (C_4_AF), wustite (FeO), and srebrodolskite (C_2_F). The most common mineral phases in EAF steel slag are larnite (C_2_S), wustite (FeO), magnetite (Fe_3_O_4_), gehlenite (C_2_AS), and quartz (SiO_2_). The C_2_S, C_3_S, and C_4_AF crystal size in steel slags is larger than in cement, and the content is much lower [23]. Hydrated inert mineral phases in steel slag, such as γC_2_S, wustite, gehlenite, and CaO-FeO-MnO-MgO solid solution, cannot form new products in the hydration process, and they only participate as fillers [28]. BOF and EAF steel slags have no pozzolana properties, and only CaO, C_3_S, and C_4_AF can react with water and show less hydration activity [33]. Calcite (CaCO_3_), portlandite (Ca(OH)_2_), and brucite (Mg(OH)_2_) have also been reported in the previous literature, and these mineral phases are mainly formed during the aging process of steel slag [34,35].

**Table 1:** Chemical compositions of steel slags in the latest literature.

Type	Year	References	Chemical Composition									
			CaO	SiO_2_	Fe_x_O_y_*	Al_2_O_3_	MgO	P_2_O_5_	Mn_x_O_y_*	SO_3_	TiO_2_	Na_2_O + K_2_O
BOF	2022	[36,37,38,39,40,41]	36.53–42.80	10.56–19.62	16.85–24.23	3.05–7.00	2.01–8.41	1.15–2.77	1.84–3.90	0.5–0.79	0.5–1.01	0.15
BOF	2021	[20,21,22,23,42,43,44,45,46,47,48,49]	36.6–43.48	8.8–18.9	16.64–32.2	1.78–9.84	3.64–10.6	0.65–2.5	2.53–5.4	0.2–1.74	0.2–1.1	0.1–0.6
BOF	2020	[50,51,52,53,54,55,56]	37.6–49.66	9.86–17.4	8.5–27.3	1.4–9.3	2.51–9.2	0.05–2.32	0.4–4.8	0.22–1.1	0.3–0.68	0.04–0.61
BOF	2019	[30,57,58,59,60,61,62]	41–50.7	9.45–20.92	14.80–27.03	0.75–5.33	2.3–10.11	1.73–2.11	1.35–4.62	0.03–0.52	0.41–1.23	0.08–0.67
BOF	2018	[63,64,65,66,67,68]	38.5–45.17	11.08–18.46	15.57–29.55	1.01–5.37	4.67–9.95	1.15–1.69	0.42–4.39	0.01–0.75	0.45–2.44	0.09–0.58
EAF	2022	[41,69,70,71,72]	24.53–55.21	12–22.42	4.4–46.74	3.01–12.6	3–8.5	0.03–0.5	0.33–5	0.143–0.4	0.22–6.16	0.12–3.04
EAF	2021	[23,26,48,73,74,75,76,77,78,79,80]	24.53–35.62	10.89–20.29	16.5–36.06	4.07–9.16	1.99–12.4	0.56–1.1	0.84–6.28	0.03–0.36	0.16–0.78	0.05–0.66
EAF	2020	[27,81,82,83,84,85,86,87,88,89,90,91]	19.4–51.23	8.59–21.99	16.78–38.7	1.29–12.2	2.97–7.53	0.311–1.52	0.3–7.9	0.205–0.94	0.441–1.02	0.31–1.51
EAF	2019	[61,92,93,94,95,96,97]	27–37.96	14.56–19.1	25.8–34	4.25–13.7	2.5–7.62	1.08–1.83	2.45–5.4	0–0.69	0.04–1	0.16–0.29
EAF	2018	[15,32,68,98,99,100,101,102,103]	22.5–38.86	9.06–20.3	22.3–35.4	3.59–15.1	3–7.72	0.2–1.5	0.48–7.35	0.42–0.74	0.38–2.11	0.13–1.7
BOF	Occurrence range		36.53–50.7	8.8–20.92	8.5–33	0.75–9.84	2.01–10.6	0.05–3	0.4–5.4	0.01–1.74	0.2–2.44	0–0.67
EAF			19.4–55.21	8.59–22.42	4.4–46.74	1.29–15.1	1.99–12.4	0.03–1.83	0.3–7.9	0–0.94	0.04–6.16	0.05–1.7
BOF	Average values		41.68	14.99	23.66	3.70	5.95	1.74	3.12	0.40	0.78	0.26
EAF			30.88	16.77	29.26	7.75	5.09	0.65	3.79	0.92	0.36	0.49

Fe_x_O_y_* refers to Fe, FeO, and Fe_2_O_3_. Mn_x_O_y_* refers to MnO and Mn_3_O_4._

### 2.3. Physical and Mechanical Properties of Steel Slag Aggregate

Table 2 shows the physical and mechanical indexes of SSA, basalt, granite, and limestone in the latest literature. Owing to the high content of metal oxides, especially ferrite and manganese oxides [104], the specific gravities of SSA commonly exceed 3000 kg/m^3^, which is 10–50% higher than that of NA. According to Table 1, the content of ferrite and manganese oxides in the EAF steel slag is higher than that in the BOF steel slag; thus, the specific gravity of the EAF steel slag is slightly higher than that of the BOF steel slag.

**Table 2:** Physical and mechanical properties of steel slag aggregate in the latest literature.

Type		Physical and Mechanical Properties						References
		Specific Gravity (kg/m^3^)	Los Angeles Abrasion (%)	Crushed Value (%)	Polished Value (%)	Needle Flake Content (%)	Water Absorption (%)	
BOF	Occurrence range	3000–3750	11.5–19.6	3.6–14.4	49–62	5.9–9.8	1.07–3.45	[36,38,45,48,56,57,105,106,107]
	Average values	3370	15.84	7.96	54	7.81	2.12	
EAF	Occurrence range	3000–3900	13.3–25.9	5.93–24.26	N/M *	0–9.42	0.922–2.93	[15,27,32,68,73,82,83,84,85,86,87,90,93,94,95,96,97,98,99,100,101]
	Average values	3530	17.93	14.54	N/M *	3.12	1.94	
Basalt	Occurrence range	2500–3000	8–14	5–12	45–55	Requirement in China ** ≤8 or 15 or 25	0.3–1.5	[57,82,83,85,87,93,97,104,108,109,110]
Granite	Occurrence range	2400–2800	18–22	19–24	45–56		0.2–1.2	
Limestone	Occurrence range	2500–2800	21–30	20–25	N/M *		0.3–2	

N/M *: Not mentioned. Requirement in China. **: Depending on the strength grade of concrete (i.e., ≤C30, C30~C55, ≥C60).

**Figure 4:**
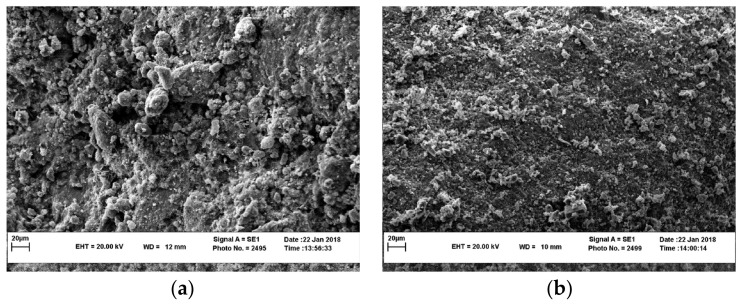
SEM images of EAF steel slag and limestone [83]. (**a**) EAF steel slag. (**b**) Limestone.

SSA has favorable mechanical properties, including strong crushing and shear strength and high resistance to abrasion and impact. Its crushing, Los Angeles abrasion value, and polishing values are comparable to basalt gravel and superior to granite and limestone gravel (Table 2). SSA has sub-angular to angular particles and low contents of needle-like particles, as shown in Figure 3. These features hinder the particles from breaking under high stress. Besides, given the honeycomb pores and small “dust” on the surface of SSA (Figure 4), the surface texture of its particles is much rougher than that of NA [83,111].

The water absorption of steel slag particles is higher than that of NA, and the average water absorption values are 2.12% and 1.94%, respectively. The non-interconnected pores in steel slag provide storage space for water. The study conducted by Sun et al. [44] reported that the water absorption of BOF steel slag is higher than that of limestone aggregate, but the absorption process was relatively slow. The BOF steel slag needed 24 h to reach the saturated state, whereas limestone only required 8 h.

## 3. Volume Stability of SSA

### 3.1. Expansion Mechanism

As shown in Table 1, a large amount of CaO and MgO exist in steel slag, and their content in BOF and EAF steel slag exceeds 40% and 30% by mass, respectively. During the cooling process of steel slag, most of the CaO and MgO have been mixed with other metal oxides to form a solid solution, losing their hydration activity [28]. However, a small amount remains as free CaO (f-CaO) and free MgO (f-MgO). The content of f-CaO in BOF steel slag is about 2–10%, whereas that in EAF steel slag is about 0.5–2% [19,21,36,42,50,57,82,112]. The hydration of f-CaO and f-MgO is accompanied by harmful volume expansion, reaching 98% and 148%, respectively [113]. The hydration of f-MgO is difficult in high basicity media, so the expansion caused by f-MgO only occurs after the basicity of the slag has decreased, usually taking several years. The transition of highly active β-C_2_S to less active γ-C_2_S can also drive the expansion of steel slag, and the volume increase is only about 12% [114]. Therefore, the hydration of f-CaO is the main reason for the volume expansion of steel slag.

**Figure 5:**
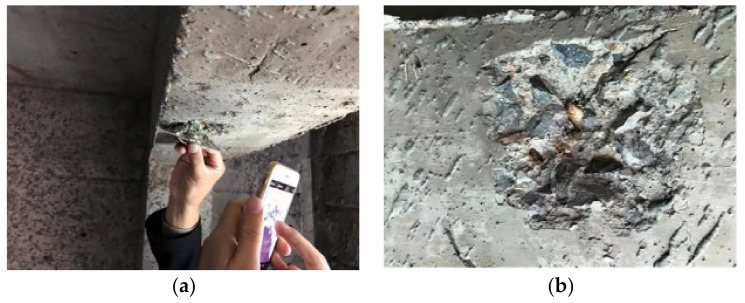
Cracking and spalling of concrete caused by the expansion of SSA [14]. (**a**) Severe cracks in engineering. (**b**) Details of cracks.

**Figure 6:**
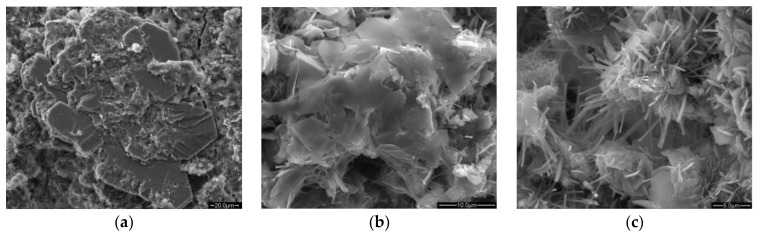
Morphology of calcium hydroxide and ettringite in natural aggregate concrete (NAC) and SSAC [115]. (**a**) Ca(OH)_2_ in NAC. (**b**) Ca(OH)_2_ in SSAC. (**c**) Ettringite in SSAC.

The aggregate is tightly embedded in concrete, with little room for free expansion. The local stress caused by the expansion of SSA can crack concrete easily, or the concrete structure could even suddenly burst during service. The specific crack morphology is shown in Figure 5. The Ca(OH)_2_ produced by the hydration of f-CaO forms irregular, large massive crystals, which is significantly different from the Ca(OH)_2_ produced by the hydration of cement, which has a regular layered or sheet-like structure [115]. Simultaneously, a large amount of ettringite has also been observed in concrete with expansion cracking (see Figure 6), which is uncommon in ordinary concrete. The production of Ca(OH)_2_ crystals causes the concrete to crack, providing enough room for ettringite growth. The growth of ettringite further promotes the expansion of cracks, allowing more water to enter the concrete, and further aggravates the expansion of steel slag. The continuous generation of Ca(OH)_2_ and ettringite crystals eventually cause cracking and spalling of the concrete.

### 3.2. Treatment Methods

Steel slag produced by steel mills must be preliminarily treated for a high cooling rate. Common preliminary treatment methods include water spray, water quenching, air quenching, and instantaneous slag chill [116]. These methods are quite mature and widely put into practice. In the preliminary treatment process, steel slag is broken into small particles under temperature stress or external force, saving the multi-stage crushing equipment [117]. Unfortunately, steel slag cannot be directly used in concrete after the preliminary treatment and requires further treatment to ensure volumetric stability [104]. Therefore, the latest post-treatment methods of steel slag volume stability are reviewed as follows.

#### 3.2.1. Natural Aging

Natural aging is a traditional post-treatment option for steel slag stabilization. The steel slag discharged from the steel mill is stored in the slag disposal yards and relies on rain and moisture in the air to consume f-CaO and f-MgO. Natural aging does consume a certain amount of f-CaO and f-MgO in the early stage, but it also leads to structural densification of the steel slag surface and prevents further reaction [20,40]. Chen et al. [118] tested the content of f-CaO in steel slag by chemical titration, and the results showed that the average content of f-CaO in steel slag aged for one year was higher than that of fresh steel slag. There are still enrichment areas of f-CaO and f-MgO in the steel slag after natural aging treatment, and it is not easy to achieve the expected modification effect.

Therefore, natural aging is slow and time-consuming, occupying a large area of slag disposal yards to provide adequate stockpiling area [119,120].

Several techniques have been developed based on the same principle to solve the problems of prolonged time consumption, low efficiency, and poor modification quality of natural aging. Kumar et al. [121] proposed a new process to accelerate the aging of steel slag by using steam generated from steelmaking waste heat. Pressurized steam permeates into the steel slag through tiny pores and can accelerate the elimination of instability. The new process can effectively reduce the discharge of waste steam in steelmaking, which is a promising solution. Meshram et al. [122] invented a high-pressure steam aging reactor with a capacity of 100 t, and the expansion rate of the treated steel slag was only 0.5%. Moon et al. [123] proposed a method of soaking steel slag in warm water at 80 ± 3 °C and claimed that the content of f-CaO could be reduced to less than 2%.

#### 3.2.2. Accelerated Carbonation

Under high-temperature, high-pressure, and high-CO_2_ concentration conditions, CaO, MgO, and other calcium-containing minerals in steel slag rapidly form stable carbonate crystals, called accelerated carbonation [69,124,125]. Accelerated carbonation can effectively improve the volume stability of steel slag. More importantly, carbon negation can be achieved by capturing and storing CO_2_ in steel slag, effectively turning steel slag into a safe and stable carbon sink. According to different reaction conditions, the accelerated carbonation of steel slag can be divided into dry and aqueous.

**Figure 7:**
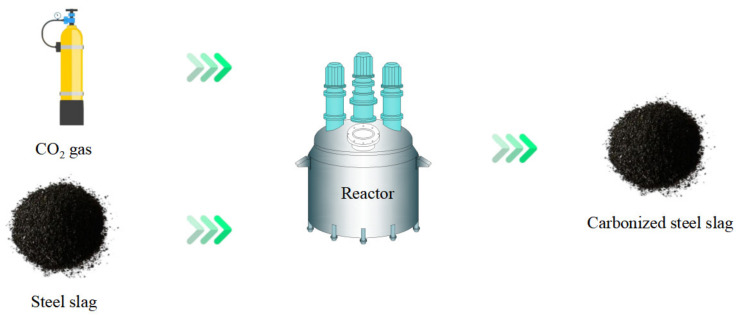
Flowchart of dry carbonation.

Dry carbonation refers to the reaction of solid steel slag with CO_2_ gas in a high-temperature and -pressure environment. The chemical processes of dry carbonation are presented in Equations (1)–(3), and the technical flow is shown in Figure 7 [126,127]. CO_2_ gas diffused into the steel slag through the vesicular pore structure. Then, the solid chemical components such as CaO, MgO, and Ca(OH)_2_ are carbonated to produce CaCO_3_ and MgCO_3_. The dry carbonation reaction can directly feed flue gas from steelmaking into the reactor to simultaneously achieve the capture of CO_2_ and stabilization of steel slag [128].

CaO_(s)_ + CO_2(g)_→CaCO_3(s)_ (1)

MgO_(s)_ + CO_2(g)_→MgCO_3(s)_ (2)

Ca(OH)_2(s)_ + CO_2(g)_→CaCO_3(s)_ + H_2_O (3)

**Figure 8:**
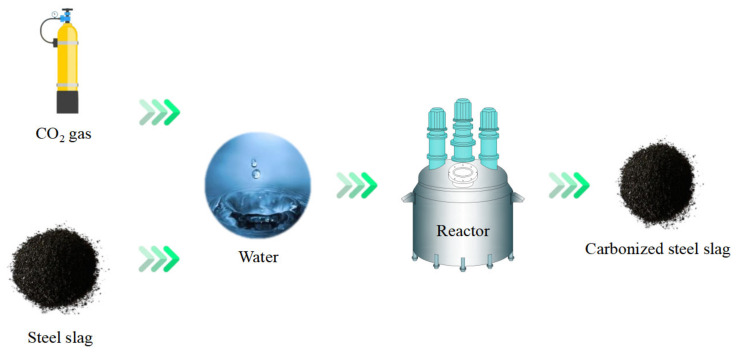
Flowchart of aqueous carbonation.

Compared with dry carbonation reaction, aqueous carbonation is a complex three-phase reaction process, which can obtain a higher reaction rate and carbonation level at a relatively low reaction temperature (still higher than room temperature) and pressure conditions [69,129]. The technical flow and chemical processes of aqueous carbonation are shown in Figure 8. First, CaO, C_2_S, and C_3_S in steel slag are leached and hydrated, and the hydration products are Ca(OH)_2_ and C-S-H. The chemical processes involved in the process are shown in Formulas (4)–(6) [40,41,43,51]. Many active carbonation components appear in the hydrated suspension, including leached C_2_S and C_3_S and hydration products Ca(OH)_2_ and C-S-H. Subsequently, CO_2_ gas is dissolved into water to form CO_3_^2−^, and CO_3_^2−^ reacts with the above active components to form C-S-H and CaCO_3_. The C-S-H is an intermediate product, which will further carbonize to form SiO_2_ and CaCO_3_ [130]. The chemical processes are shown in Formulas (7)–(9) [43,51]. The reaction of Mg-containing minerals in aqueous carbonation reaction is minimal because the leaching of Mg-containing chemical components is slow, and the carbonation products of Ca-containing minerals block the leaching channel of Mg-containing minerals [40,43].

CaO + H_2_O→Ca(OH)_2_ (4)

3CaO·SiO_2_ + (3 + y − x)H_2_O→xCaO·SiO_2_·yH_2_O + (3 − x)Ca(OH)_2_ (5)

3CaO·SiO_2_ + (2 + y − x)H_2_O→xCaO·SiO_2_·yH_2_O + (2 − x)Ca(OH)_2_ (6)

Ca(OH)_2_ + CO_2_→CaCO_3_ + H_2_O (7)

3CaO·SiO_2_ + yH_2_O + (3 − x)CO_2_→xCaO·SiO_2_·yH_2_O + (3 − x)CaCO_3_ (8)

2CaO·SiO_2_ + yH_2_O + (2 − x)CO_2_→xCaO·SiO_2_·yH_2_O + (2 − x)CaCO_3_ (9)

**Figure 9:**
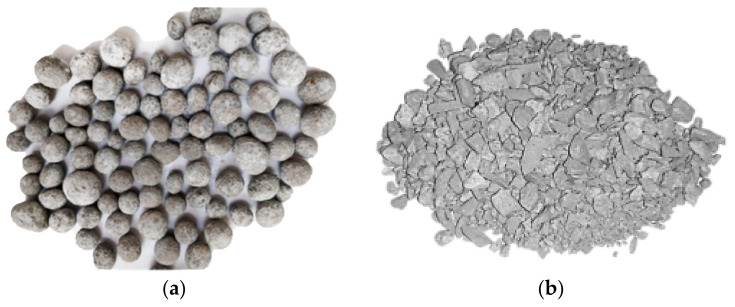
Artificial aggregate material. (**a**) Granulation aggregate [134]. (**b**) Compaction aggregate [135].

The particle size of steel slag is an important factor affecting the accelerated carbon rate and the treated quality. On the one hand, as particle size increases, the specific surface area of particles decreases correspondingly, making steel slag less reactive to CO_2_ [131]. On the other hand, the carbonization reaction gradually produces a dense, carbonized shell on the surface of steel slag particles, which hinders the leaching of active carbonation components and the diffusion of CO_2_ [132]. Therefore, the carbonation reaction decreased with an increase of the particle size. The object of accelerated carbonation in previous studies was mainly steel slag powder with a particle size of 0.05–1 mm [40,41,43,125,128]. The latest research combines accelerated carbonation techniques with artificial granulation or compaction manufacturing processes to make steel slag powder into artificial aggregates [51,133], as shown in Figure 9. The mineral phases of CaCO_3_ produced by accelerated carbonation are aragonite (dry carbonation) and calcite (wet carbonation), and their crystal hardness reaches 67 GPa and 72.8 GPa, respectively. These mineral phases can form a dense organizational structure in the steel slag [69]. Therefore, the overall properties of the artificial SSA after accelerated carbonation treatment are significantly enhanced.

Jiang et al. [51] investigated the effect of accelerated carbonization on the properties of 100% BOF steel slag artificial aggregate, and the result shows that the strength increased by 220%. Ko et al. [136] also reported the positive influence of accelerated carbonization on the mechanical properties of artificial steel slag columns, and the crushing strength of carbonized BOF steel slag columns was 38.9–69.1% higher than that of non-carbonized samples. The laboratory study results of Pang et al. [137] showed that the harmful pores in the carbonized artificial steel slag particles decreased by 24.4%, and the harmless pores increased by 67.9%. More importantly, the mechanical strength of concrete prepared with carbonized artificial SSA can be comparable to that of ordinary concrete and light aggregate concrete [134,135].

#### 3.2.3. Molten Slag Converting

The principle of molten slag converting is adding modifiers in remelted steel slag to convert its composition. Modifiers react with f-CaO and f-MgO in molten steel slag to crystallize f-CaO and f-MgO, thus improving the volume stability of steel slag. Long et al. added 5%, 7%, and 14% fly ash to molten steel slag as the modifier. After the reaction at 1580 °C for 30 min, the corresponding digestion rate of f-CaO was 56.99%, 63.69%, and 68.55%, respectively [17]. Zhang et al. added iron tailings to molten steel slag and studied the influence of iron tailings content, reaction temperature, and reaction time by orthogonal experiment. The results show that the iron tailings can effectively dissolve f-CaO in steel slag, and the digestion rate reaches 76.27% [138]. Mombelli et al. found that adding quartz sand to molten steel slag can induce the formation of gabbro, thus ensuring the inert behavior of steel slag [139].

#### 3.2.4. External Constraint

**Figure 10:**
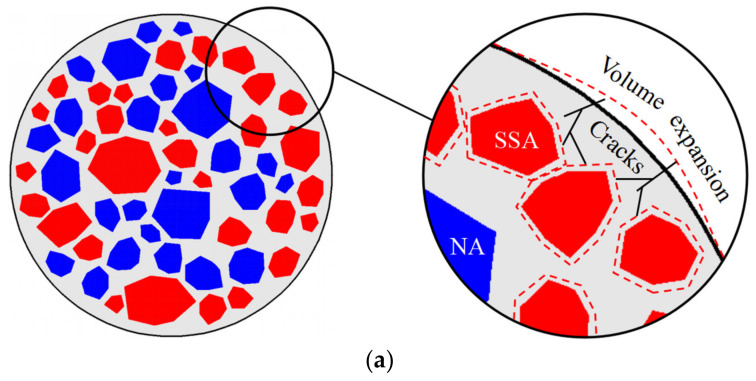

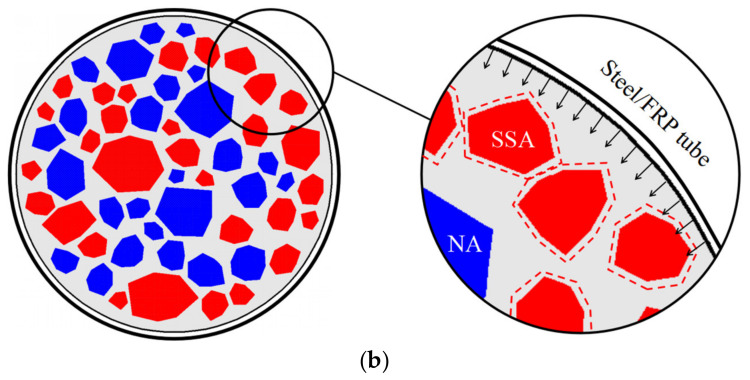
The expansion limit schematic diagram of the external constraint technique. (**a**) Cross-section of SSAC. (**b**) Cross-section of steel/FRP tube-confined SSAC.

The external restraint technique refers to confining SSAC with a steel/FRP tube to limit its cracking and spalling [140,141]. As shown in Figure 10, the high modulus and tensile strength of steel/FRP tubes can effectively limit the uncertain expansion of SSA. Simultaneously, the expansion of SSA can make the core concrete enter a three-way stress state in advance, reducing the negative influences of the interface gap effect and delaying the confining effect in the service stage. Feng et al. [14] studied the volume stability of SSAC columns confined by GFRP, and the 3D laser scanning results showed that FRP tubes could effectively inhibit the cracking and spalling of concrete caused by SSA expansion. Yu et al. [18,142] proposed a self-stressing steel slag aggregate concrete filled steel tubular stub columns and found that the expansion of the core steel slag concrete can enhance the axial compression capacity of the short column and the bond strength of the steel-concrete interface.

### 3.3. Characteristics of the Treatment Methods

Table 3 summarizes the positives, negatives, and applicability of the introduced treatment methods. At present, natural aging is the primary management method of steel slag, which has the advantages of low investment and simple operation, and its treated capacity is mainly related to the area of the slag disposal yards. The natural aging process is time-consuming, requiring a large area of land resources, but the modification quality is poor. In addition, some heavy metal oxides with significant biological toxicity exist in steel slag [22,120]. These heavy metals will spread to soil and water and accumulate in the food chain, finally resulting in severe environmental hazards. The hot steam aging process can significantly improve the modification quality of steel slag, but the whole process still takes several days [121]. Adding hot steam aging equipment to the crude steel production line may reduce production efficiency.

Accelerated carbonation is a highly environmentally beneficial technique that can achieve the desired modification quality in a short period. By optimizing the temperature, pressure, humidity, CO_2_ concentration, reaction time, and steel slag particle size, the CO_2_ capture capacity of steel slag per kilogram can reach 4.7–516.9 g [16,43,51,128]. The current steel slag recycling process can fix 60–80 million tons of CO_2_ gas annually worldwide [143], thereby mitigating global warming. The accelerated carbonation technique of steel slag has many links and complicated operations. The grinding energy consumption of steel slag is 36.7 kWh/ton, and the laboratory accelerated carbonation cost of steel slag powder is 42.1 $/ton [144]. Therefore, cost control will be a key issue after this method is applied to production. So far, the market demand and total production of carbonized steel slag products are insufficient, and their contribution to reducing CO_2_ sequestration is small.

Molten slag converting technology can consume f-CaO and f-MaO quickly and improve the hydraulic and pozzolanic activity of steel slag, significantly improving the grindability of the treated steel slag [145]. However, this modification technique can only apply to molten steel slag and requires high fluidity of steel slag. If this technology is used to treat solid steel slag, remelting will cause additional energy consumption.

Filling SSAC into steel/FRP tubes is an effective and easy application that could accept the uncertain expansion of SSA. In particular, there are no additional processing and usage limits for SSAs, which would be more attractive for construction units. The use of steel/FRP tubes may cause an increase in material costs. However, the initial cost may be balanced, or even outbalanced, by the reduction in maintenance cost throughout the service life due to the excellent corrosion resistance of the jacket [146]. Besides, the confinement of steel/FRP tubes can offer a level of enhancement hardly achievable by other methods in both strength and deformability of the core concrete, thus reducing the excess self-weight caused by high-density SSA.

Overall, the introduced treatment methods can consume f-CaO during the total life cycle of steel slag. In the hot slag stage, hot steam aging equipment and molten slag converting equipment can be installed on the steel slag production line to treat the fresh steel slag according to its characteristics. For the steel slags stockpiling in slag disposal yards, f-CaO content and expansion risk should be tested first. Then, an assessment should focus on the proposal for further treatment. Steel slags with qualified volume stability can be directly used in the production of SSAC. For the steel slag that still has the threat of uncertain expansion, it can be considered to make carbonized artificial SSA or used for steel/FRP tube-confined SSAC composite structures. In this process, combining multiple technologies should be tried to balance the capacity and cost of steel slag modification. Research on pilot and industrial trials to connect these methods as a new complete system needs to be further conducted.

## 4. Utilizing Treated SSA in Concrete Production

Over the years, uncontrolled concrete production has led to the overexploitation of high-quality river sand and gravel, leading to severe concerns about resource depletion. Utilizing treated steel slag as coarse/fine aggregate in concrete production is a high-value solution that can alleviate the worry about resource depletion and solve the negative impact of steel slag stockpiling in slag disposal yards.

### 4.1. Density

**Figure 11:**
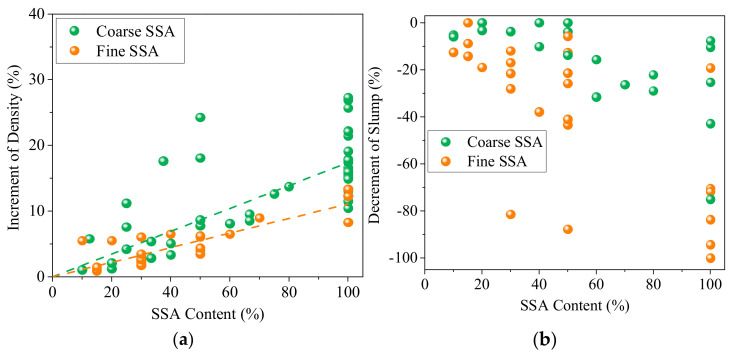
Variation of the density and slump of concrete at different SSA content [32,36,48,72,80,86,87,93,95,96,98,113,149,150,151,152,153,154]. (**a**) Density. (**b**) Slump.

The density of SSA is 10–50% higher than that of NA. When designing the mix proportion, the amount of SSA should be calculated by the same volume, with the NA being replaced, but not of the same weight. Figure 11 collects the variation of concrete density for different SSA content, and using SSA could increase concrete density by up to 30%. The density increment rate of concrete depends on the content and type of SSA, and coarse SSA has a higher improvement than fine SSA. Therefore, using SSAC in a building structure should take into account the excess self-weights. To guide the self-weight estimation of SSAC, the data collected in the literature are fitted, and the results are presented in Formulas (10) and (11). Where *D_c_* refers to the density of concrete prepared with coarse SSA, *D_f_* refers to the density of concrete prepared with fine SSA, and *r* stands for the replacement ratio of SSA. The R^2^ of Formulas (10) and (11) are 0.49 and 0.68, respectively, caused by the difference in the concrete mix ratio and the density of selected steel slag in the literature.

*D_c_* = 0.1736*r* (10)

*D_f_* = 0.1109*r* (11)

In addition, the increase in density means SSAC could be potentially used as a shielding material against the nuclear radiation of X-rays, gamma rays, and neutrons. In work conducted by Baalamurugan [147], the linear attenuation coefficient of ^60^Co in concrete increased by 6.6% and 14.5%, respectively, when SSA replaced 25% and 50% of coarse NA. Pomaro et al. observed that, by substituting 100% coarse SSA for NA, the half-value layer of ^60^Co and ^137^Cs increased 12~16.8% and 15.7~17.8%, respectively [95]. In contrast, the half-value layer of SSAC is 9.5% higher than barite concrete and 10% lower than hematite concrete [148]. Utilizing SSA to produce radiation-shielding concrete could reduce production costs and avoid overutilizing natural resources (e.g., hematite, magnetite, barite, and serpentine).

### 4.2. Workability

Because SSA has a higher water absorption than NA, using SSA in concrete reduces the amount of free water in the mix. In addition, the surface texture of SSA is rougher than NA due to its honeycomb pores and small “dust” on the surface, which increases the frictional resistance between particles during the flow of concrete paste. Therefore, the fluidity, cohesiveness, and water relativity of the SSAC mixture are generally poor. Figure 11b collects the slump of concrete at different SSA content, and the slump of fresh concrete decreases smoothly with the increase of SSA usage. Replacing 100% river sand or gravel with SSA without any mix ratio adjustment can reduce the slump by 75% and 100%, respectively [80,149]. It is obvious that fine SSA weakens the workability of fresh concrete more significantly than coarse SSA. The amount of superplasticizer or filler (cemented or inert) should be increased to maintain a qualified slump [36,87] or adjust water content according to the water absorption of SSA [96].

### 4.3. Mechanical Properties

**Figure 12:**
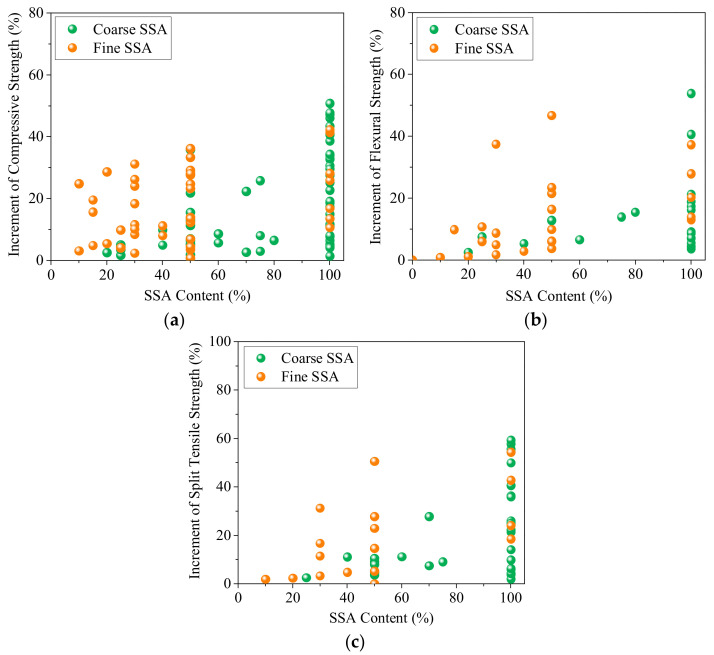
Increment of the mechanical strength of concrete at different SSA content [26,36,48,72,74,86,87,89,93,95,96,98,147,151,152,155]. (**a**) Compressive strength. (**b**) Flexural strength. (**c**) Split tensile strength.

Using SSA can improve the mechanical strength of concrete. Figure 12 summarizes the mechanical properties of concrete at different SSA content. SSAC had superior mechanical strength compared to natural aggregate concrete (NAC), and higher SSA content leads to higher strength increments. SSA can maximum enhance the compressive, flexural, and split tensile strength of ordinary concrete by up to 50%, 60%, and 60%, respectively. Fine SSA has a more significant strength enhancement effect than coarse SSA, which is mainly due to the positive influence of the active mineral phase components in the powder on the hydration degree of the mixture [113]. However, the highest fine SSA dosage is usually 50% due to an increased loss of working performance. Some negative results are mainly due to the poor workability and cracking caused by the volume expansion [15,153].

The improvement in mechanical strength of concrete can be attributed to the following aspects: (a) greater contact area between mortar and SSA. The vesicular nature and rough surface texture of SSA increase the contact area with the mortar (see Figure 3 and Figure 4); (b) A stronger interfacial transition zone (ITZ). The high water absorption of SSA reduces the local water-binder ratio in the concrete, and the active mineral phase components on the surface of SSA can increase the hydration degree of the mixture. The enhancement mechanism of ITZ includes strength improvement, width reduction, and structural densification [156]; (c) SSA has better mechanical properties than NA. The strong crushing and shear strength and high resistance to abrasion and impact of SSA can prevent the crushing, breaking, and wearing of particles at high-stress levels [24]. Using SSA in concrete production can appropriately reduce the amount of cement due to the increase in mechanical strength, which helps to alleviate the resource and environmental dilemma caused by cement production.

In addition, the elastic modulus of SSAC is higher than that of NAC. Pomaro et al. [95] reported that a mixture of 100% coarse EAF aggregate could increase the secant elastic modulus by up to 33.9%. Liu et al. [157] prepared porous cement concrete with 100% coarse SSA and observed a significant increase in elastic modulus at 61.5%. Beaucour et al. [86] compared concrete with coarse aggregate from EAF steel slag, barite, and dolomite, while SSAC has a dynamic modulus of 50.1% higher than barite concrete and 16.8% higher than dolomite concrete. Modulus improvement of SSAC is also due to the enhanced ITZ and the superior mechanical properties of SSA [95].

### 4.4. Durability of SSAC

Replacing NA with SSA harms the water tightness of concrete. Substituting 50% to 100% coarse SSA for NA, the total water absorption could be maximized by 13% and 24% [23], and the water penetration depth increased smoothly to 6% and 25% [74]. The volume of permeable voids of SSAC is 30% higher than NAC due to the pores in SSA providing excess water storage space [158]. Reducing the angularity of SSA or using fly ash can improve the watertightness of concrete [32,159].

Compared with the features of NAC, the frost resistance durability of SSAC is more satisfactory. On the one hand, the rough texture of SSA can activate a stronger interlocking effect in ITZ, resulting in a closer bond between SSA and cement mortar [59,160]. On the other hand, the pores and cavities in the SSA can alleviate the swelling force caused by water freezing [93]. Therefore, after the same freeze-thaw cycles, SSAC can maintain better integrity and higher mechanical strength than NAC. Chatzopoulos et al. [26] performed 14 freeze-thaw cycles on NAC and SSAC (30% fine SSA and 50% coarse SSA) samples. After the freeze-thaw test, the edges of the NAC samples rounded, but the SSAC samples kept a square shape. The weight reduction of SSAC samples is 3.5 times smaller than that of NAC samples. In the study conducted by Wu [153], after the same freeze-thaw cycles, the mass loss, compressive strength loss, and flexural strength loss of the SSAC decreased by 76.3%, 83.4%, and 13%, respectively, compared to the control concrete. Sosa et al. [72] conducted freeze-thaw cycle tests on NAC and SSAC samples in 5% NaCl solution, and the results showed that the limit number of freezing-thawing cycles of NAC samples was 7, and that of SSAC samples was 21.

Utilizing SSA has a beneficial effect in improving the chloride resistance of concrete. The conductivity of SSA is higher than that of NA, which reduces the overall resistivity of concrete and results in a higher total charge pass (TCP) of SSAC [155]. However, the final chloride penetration depth in SSAC is smaller than NAC due to the dense ITZ between SSA and mortar limiting chloride penetration. Chatzopoulos et al. demonstrated that using SSA in concrete reduces chloride infiltration by up to 57% [26]. When exposed to sulfate environments, the inclusion of SSA slightly reduced the durability performance of concrete. The ITZ will be weakened due to the CaCO_3_ present on the surface of the SSA reaction with MgSO_3_ solution to form gypsum [158]. Adding mineral mixtures can significantly improve the resistance to sulfate corrosion of SSAC, with fly ash showing better performance [15].

Concrete produced with SSA presents better carbonation resistance. Chatzopoulos et al. [26] reported that the CO_2_ penetration of SSAC is 55% lower in natural carbonation and 40% lower in accelerated carbonation than NAC. Andrade et al. [48] designed three types of concrete with strength grades 15 MPa, 25 MPa, and 35 MPa and replaced NA with BOF and EAF steel slag, respectively. The test results show that the carbonization depth of SSAC is 80% lower than that of NAC. The high-alkalinity pore solution of SSA can inhibit the carbonation reaction in concrete [49,161]. Simultaneously, the carbonization of SSA forms high-density calcite in ITZ, which causes the concrete to be densified and hinders the permeability of moisture and CO_2_. Therefore, SSA has a lower carbonation depth in the same accelerated carbonation period than NAC.

## 5. Economic and Environmental Benefits of Using SSA

Although the volumetric expansion treatment brought additional costs, SSA is still highly economical as a sustainable building material. The price of SSA is only about 20% that of NA [144]. Shen et al. prepared pervious concrete with carbonated SSA, which could decrease costs by 77.8% and decrease 100 kg of CO_2_ emissions per cubic meter [162]. In addition, using SSA can improve the mechanical strength and durability of concrete, and the economic benefits are also brought by reducing cement consumption and maintenance costs. The cost-effectiveness of steel slag makes it a viable candidate in concrete industry applications.

The utilization of SSA in the concrete industry can effectively reduce the exploitation of high-quality river sand and gravel, thus alleviating growing concerns about resource depletion and environmental pollution. Simultaneously, the consumption of steel slag can release many occupied land resources and alleviate the conflict between the steel industry and land. In addition, some heavy metal oxides with significant biological toxicity exist in steel slag; Mn, Zn, Cr, Cd, Ni, Al, V, and Pb are examples that can be given [39]. If steel slag is stockpiled in slag disposal yards, heavy metals will spread to the soil and water, resulting in severe environmental hazards. Cement-based materials can play an essential role in solidifying heavy metals through adsorption, forming insoluble substances, substituting ions or ionic groups, physical encapsulation, and other ways [163,164,165,166]. Therefore, using SSA in concrete can reduce the leaching concentration of heavy metals. Normal concrete, with a 20–30% coarse SSA replacement rate, has been proven to entail good environmental safety [167,168,169], and further research is ongoing. In ultra-high-performance concrete, there is a non-detectable leaching of pollutants when the replacement rate of SSA reaches 100% [170,171].

## 6. Conclusions

In the present article, the production, properties, and treatment method of SSA and the performance of SSAC were reviewed. Steel slag is the main solid waste of crude steel smelting, and the main chemical components are oxides of Ca, Fe, Si, Al, and Mg. The cementitious mineral in steel slag belongs to the crystalline phase, which has poor hydraulic activity but no pozzolanic activity. Steel slag particles have the characteristics of high-density, rough surface texture, and good mechanical properties, their specific gravity is 10–50% higher than that of NA, and their mechanical strength is comparable to basalt gravel and superior to granite and limestone gravel. Therefore, steel slag particles have the potential to be used as coarse/fine aggregate in concrete production.

Steel slag is still a solid waste rather than an available resource because of the poor volume stability of SSA. The expansion chemical components in steel slag include f-CaO, f-MgO, and β-C_2_S. Among them, f-CaO is the main reason for the volume expansion of steel slag. The harmful effects of using untreated steel slag directly as an aggregate are obvious and could cause cracking and spalling of concrete. Therefore, SSA must be treated to control the content of f-CaO. The methods used to modify the poor volume stability of SSA include natural aging, hot steam aging, accelerated carbonization, molten slag converting, and external constraint. These methods have made significant progress and have, partly, been put into practice. The advantages, disadvantages, and applicability of treatment methods in making steel slag treatment schemes should be taken into account, and different methods should be combined.

Replacing NA with SSA could increase concrete density. Thus, SSAC is a type of heavy-weight concrete potentially used as a shielding material for nuclear radiation of X-rays, gamma rays, and neutrons. Replacing 100% river sand or gravel with SSA can reduce the slump by 75% and 100%, respectively. In the design of concrete mix ratio, the amount of superplasticizer or filler (cemented or inert) should be adjusted according to the SSA type and content. Fine aggregate is not recommended to replace more than 50% ratio with fine SSA due to a serious deterioration in the workability of concrete, and the coarse SSA could replace NA at ratios up to 100%. Ongoing studies show that, thanks to ITZ enhancement and the superior mechanical properties of SSA, well-designed SSACs commonly show superior mechanical strength to NAC, and the compressive, flexural, and split tensile strength can be improved by up to 50%, 60%, and 60%, respectively.

Using SSA in concrete can provide great economic, environmental, and sustainable development benefits. SSAC showed lower total lifecycle costs. The price of SSA is only 20% that of NA, which can significantly reduce concrete production costs. The water tightness of SSAC is poor, but it still has good resistance to freeze-thaw cycles, corrosion, and carbonation, which is an outstanding advantage of utilizing steel slags as coarse/fine aggregate without decreasing the durability of concrete. Using SSAC allows a longer service life of structures and saves maintenance costs during service life. In addition, the consumption of steel slag can reduce slag disposal yard occupation and environmental pollution risk and avoid over-exploitation of high-quality sand and gravel. Meanwhile, the acceptable leaching results indicate that SSAC showed adequate environmental safety. This case implies a perfect industrial-ecological relationship between the steel industry and the concrete industry for achieving sustainable development.

## 7. Future Research Needs

The analyses of existing works indicate that steel slag has the potential to be utilized as aggregates for concrete. However, many research gaps in this field need further study, thus providing more critical evidence for utilizing steel slag. On the basis of the literature review, the following recommendations for future research are forwarded:

(1) Combining artificial granulation and accelerated carbonization is a promising method for steel slag modification with good quality and high environmental benefits. However, this technique is still in the laboratory research stage. Future research should focus on simplifying the production process, increasing production capacity, and controlling production costs to realize the industrial production of carbonized artificial SSA.
(2) Filling SSAC into steel/FRP tubes can limit the cracking and spalling of concrete caused by the expansion of SSA, and the expansion of SSA can reduce the negative impact of the interfacial gap effect in the service stage; such composite structures are promising. In future research, an assessment should focus on the mechanical properties and design model of steel/FRP tube-confined SSAC composite structures.
(3) Different sources are inconsistent in reports on the performance of steel slag. Mix proportions and properties of concrete prepared with SSA from one source cannot be extended to all sources, which hindered the research and large-scale usage of SSA. Conducting a global data survey on the SSAC and creating a primary dataset is necessary for future research. Then, this primary dataset should be used to train an artificial neural network to predict the properties of SSAC.
(4) The research on the mechanical properties of SSAC mainly focuses on cube compressive strength, flexural strength, and flexural strength. In the future, research should be carried out on the fracture mechanical properties and deformation ability of SSAC, and the bond properties between SSAC and steel bars, to provide data support for the application of SSAC in broader fields.
(5) There is a gap in research on the heavy metal leaching behavior in SSAC, and works have only been carried out in the condition of a low, coarse SSA replacement ratio. Future research should be conducted for more complex situations, including different SSA types, different SSA particle sizes, and higher SSA contents, to guide the decision of the SSA utilization scheme.

## Figures and Tables

**Table 3:** Positive and negative characteristics of introduced treatment methods.

Techniques	Advantages	Limitations	Applicability
Natural aging	(1) Low costs (2) Easy operation (3) High modificationcapacity	(1) Vast slag disposal yards (2) Long period (3) Poor treated quality (4) High environmental hazard	All types
Hot steam aging	(1) High treated quality (2) High environmental benefits	(1) Long period (2) High requirements on equipment	All types
Accelerated carbonation	(1) High treated quality (2) High environmental benefits (3) Short period	(1) High costs (2) Low modification capacity (3) High requirements on equipment (4) High technical difficulty	PowderArtificial aggregate
Molten slag converting	(1) High treated quality (2) Short period (3) Low costs	(1) High requirement on the fluidity of slag (2) High requirements on equipment	Molten steel slag
External constraint	(1) Easy operation (2) No additional processing (3) High-volume stability	(1) High material costs (Steel/FRP tube) (2) Good mechanical properties	All types

## Data Availability

Data available on request due to restrictions, e.g., privacy or ethical.
